# Optimal Serum 25(OH)D Levels and Vitamin D Intake in Korean Postmenopausal Women

**DOI:** 10.3390/nu15081856

**Published:** 2023-04-12

**Authors:** Hye Ran Shin, Ye Jin Lee, Sun Yung Ly

**Affiliations:** Department of Food and Nutrition, Chungnam National University, Daejeon 34134, Republic of Korea; hyeran@cnu.ac.kr (H.R.S.); lyj@o.cnu.ac.kr (Y.J.L.)

**Keywords:** Korean postmenopausal women, optimal serum 25(OH)D levels, optimal vitamin D intake

## Abstract

Vitamin D plays a crucial role in regulating the growth and maintenance of the musculoskeletal system. Postmenopausal women are vulnerable to bone fractures because of the decrease in bone mineral density (BMD). Therefore, this study aimed to identify the determinants that influence BMD and the 25(OH)D levels in Korean postmenopausal women. This study collected general and dietary intake information, measured biochemical indices, and conducted BMD tests in 96 postmenopausal women residing in a metropolitan area in Korea. This study analyzed factors that influenced serum 25-hydroxyvitamin D (25(OH)D) and BMD, as well as the correlation between the intact parathyroid hormone (iPTH) and serum 25(OH)D levels. The serum 25(OH)D levels increased by 0.226 ng/mL in the summertime, 0.314 ng/mL in the wintertime, and 0.370 ng/mL on annual average when vitamin D intake rose by 1 µg/1000 kcal. When the serum 25(OH)D levels were ≥18.9 ng/mL, the iPTH levels did not rapidly increase. To maintain the serum 25(OH)D levels at ≥18.9 ng/mL, a daily vitamin D intake of ≥13.21 µg was required. Consequently, consuming vitamin D-fortified foods or vitamin D supplements is necessary to improve both bone health and vitamin D nutritional status.

## 1. Introduction

Vitamin D is a fat-soluble prohormone affecting the expression of more than 200 human genes [1]. Although it is a classically essential vitamin for bone health, vitamin D has also been related to osteoporosis, bone metabolism, and the risk of bone fractures [2]. Recently, numerous studies have emphasized the importance of vitamin D in preventing depression, cardiovascular disease, diabetes, cancer, and immune disorders [2,3,4], making it an essential nutrient throughout life. Unfortunately, more than a billion individuals worldwide are estimated to experience vitamin D insufficiency [5], while over 200 million people are affected by osteoporosis [6].

Vitamin D plays a vital role in preventing bone fractures, especially in postmenopausal women [7,8]. Lower estrogen levels after menopause lead to increased activation of osteoclasts resulting in a substantial decrease in bone mass [9]. Vitamin D increases the intestinal absorption of calcium and phosphate, which helps maintain the mineralization of healthy bone tissue [2]. Additionally, it encourages bone resorption by increasing osteoclast activity and regulating serum concentrations of parathyroid hormone to maintain serum calcium levels [10].

Approximately 64% of postmenopausal women were reported to have serum 25(OH)D levels <30 ng/mL worldwide [11]. Because aging decreases 7-dehydroxycholesterol synthesis in the epidermis, the vitamin D concentrations of individuals in their 70s are known to be decreased by over four-fold than those of individuals in their 20s [12]. However, recent Korean investigations have found that serum 25(OH)D levels were higher in older women as compared to those of young women [13,14,15]. This phenomenon has also been reported in other Asian countries, such as Thailand [16] and Japan [17]. It is expected to occur because young adults tend to work indoors while older adults tend to work outdoors as the economies of East and Southeast Asian countries have developed [18]. According to a recent study, an age increase of 10 years resulted in a 13% reduction in vitamin D synthesis. However, no significant differences in the serum 25(OH)D levels based on sun exposure were detected between the younger and older cohorts [19]. Few studies have analyzed the factors that increase the serum 25(OH)D levels in women aged ≥ 50 years in East Asian countries, including Republic of Korea.

Vitamin D can be acquired when synthesized under the epidermis, which is exposed to ultraviolet (UV) radiation, or from diet. In mammalian epidermis, the intermediate of the cholesterol biosynthesis process, provitamin D3 (7-dehydrocholesterol), is transformed into previtamin D3. Vitamin D3 (cholecalciferol) is produced from previtamin D3 through a thermal isomerization process that is dependent on temperature [20]. However, dietary vitamin D intake is essential when vitamin D synthesis is reduced due to various factors, such as a lack of sunlight exposure due to prolonged indoor activity [21], high latitudes [22], and seasons with low sunlight [13]. In Republic of Korea, fatty fish is the major natural vitamin D dietary source, and the contribution of other source foods is inadequate [13]. Therefore, the mean daily vitamin D intake has been reported to be poor [13,14]. The adequate intake (AI) level of vitamin D, according to the dietary reference intakes for the Korean population in 2020 (KDRIs), is 10 µg/day for adults aged 19–64 years and 15 µg/day for adults aged ≥65 years [23]. However, these intake levels were set based on data derived mainly from other countries, and studies confirming the domestic situation are lacking. Thus, further research on vitamin D nutritional status in Korea is necessary.

Therefore, this study aimed to identify the factors affecting bone mineral density (BMD) and the 25(OH)D levels in Korean postmenopausal women by assessing their dietary status during four seasons in a year, measuring BMD measurements twice, and analyzing biochemical indices and their results. The findings will provide information on levels of optimal vitamin D intake, which can serve as basic data to establish the optimal vitamin D AI levels for the Korean population.

## 2. Materials and Methods

### 2.1. Study Participants

This study included postmenopausal women who had undergone menopause over 1 year ago and resided in a metropolitan area in Republic of Korea. An online community bulletin board was used to recruit participants. The inclusion criteria were as follows: postmenopausal women aged 45–69 years who had undergone menopause over 1 year ago; women who were neither underweight nor obese based on the body mass index (BMI) status (18.5 ≤ BMI < 30 kg/m^2^); women who had at least two regular meals per day; and women who did light exercise, such as walking, for at least twice or thrice a week for a minimum of 10 min. The exclusion criteria were as follows: women with chronic diseases, bone diseases, or endocrine diseases; women who had been continuously treated with drugs, excluding dietary supplements; women receiving hormone replacement therapy; women who were diagnosed with thyroid disease; and women who had undergone oophorectomy. The investigation was performed between September 2021 and August 2022. Women who consented to participate in the study did so for 1 year. The investigation was carried out in every season, including spring (March–May), summer (June–August), autumn (September–November), and winter (December–February). However, for the purpose of analysis, this research grouped the data collected in summer and autumn into “summertime” and the data from winter and spring into “wintertime.” When recruiting participants, 130 women met the eligibility criteria, but only 98 completed the study over the year, and 32 dropped out (dropout rate, 24.6%). Of the remaining participants, two women whose daily vitamin D intake was ≥100 µg, the tolerable upper intake level (UL) of vitamin D by KDRIs, were excluded, and, finally, the data of 96 participants were analyzed. This research was conducted in compliance with the Declaration of Helsinki and received approval from the Institutional Review Board of Chungnam National University (202107-SB-130-01; 27 August 2021).

### 2.2. General Characteristics, Lifestyle, and Outdoor Time

For general characteristics, data on birth and years since menopause (YSM) were investigated. For lifestyle, the drinking status (no drinking: at least 6 months of abstinence from alcohol), ways to avoid sun exposure and the number of those ways, level of physical activity, and intake of vitamin D and calcium supplements were investigated. General characteristics and lifestyle were surveyed only at the study initiation. Outdoor time was self-reported by the participants every season and included the total duration of outdoor activities during a day on weekdays and weekends in every season. Based on the data, the outdoor time between 10 a.m. and 3 p.m., when the sun’s rays were at their maximum, was estimated in minutes (min). Data on 2 days during weekdays and weekends in summertime (data from summer and autumn) and wintertime (data from winter and spring) were calculated for the average, which was considered as minutes of outdoor activities per day and utilized for the study. The participants were instructed to select multiple responses from the following choices for ways to avoid sun exposure: use of sunscreen products, such as sunscreen lotion and cosmetics; hat, mask, or parasol with UV blocking effect; or other methods. Physical activity was evaluated using the short form of the International Physical Activity Questionnaire (IPAQ) [24]. By calculating metabolic equivalents of task (MET), physical activity was divided into the following three stages. The high-intensity physical activity group comprised individuals who engaged in high-intensity physical activity for at least 3 days a week, attaining at least 1500 MET-min/week, or who participated in any combination of walking, moderate-intensity, or high-intensity activities for a minimum of 7 days a week, achieving at least 3000 MET-min/week. The moderate-intensity physical activity group included individuals who engaged in high activity for a minimum of 20 min/day on ≥3 days per week; who participated in moderate-intensity activities or walking for a minimum of 30 min/day on ≥5 days per week; or who engaged in any combination of walking, moderate-intensity, or high-intensity activities for a minimum of 5 days/week, attaining at least 600 MET-min/week. The low-intensity physical activity group (with the lowest level of physical activity) comprised individuals who did not meet the criteria for the other two groups or did not respond to this item. Regarding vitamin D and calcium supplement intake by the participants, the product name, nutrient content, daily intake, and intake frequency were investigated. The intake of supplements of vitamin D and calcium per day was assessed as follows:

nutrient content per serving ×frequency of daily intake

### 2.3. Dietary Assessment

The participants were requested to submit dietary records for 3 days, including 1 day on the weekend and 2 weekdays. Before conducting the study, a trained investigator distributed a video that introduced the dietary record method to the participants. The participants were required to self-report all their meals, including the food name and amounts of food. Concurrently, a ruler of a certain length was provided to the participants. They were instructed to put the ruler on the table when they had meals and to photograph the meal. All the photos were sent to the trained researcher, who then compared them to the participants’ self-reported dietary records. Thereafter, intake was finally confirmed and used for analysis. The Korean Nutrition Society’s CAN-Pro (5.0 web version) was used to analyze the dietary data [25]. The amount of vitamin D in foods was modified and supplemented in this laboratory and analyzed by applying the vitamin D database of the study presented in 2022 [13]. Data were entered based on the food database of CAN-Pro, but to enter the food items of the participants, the food database was modified, or foods that were not on the list were generated and used for analysis. The mean value of the nutrients for a total of 6 days for each of summertime (summer and autumn) and wintertime (winter and spring) were used for analysis.

### 2.4. Anthropometry, Biochemical Indices, and Bone Mineral Density

Anthropometry and biochemical tests of blood and urine were conducted at an examination center in Daejeon. Anthropometric measurements were taken using an automatic height measure (X-SCAN PLUS II, Jawon Medical Co., Ltd., Seoul, Republic of Korea). Subsequently, while the participants were in a fasting state, 10 mL of venous blood was collected in the morning through an upper arm vein using a serum-separating tube (BD Vacutainer; Becton Dickinson, Franklin Lakes, NJ, USA). To separate the serum, the blood in the tube was centrifuged for 10 min at 3000 rpm. Until analysis, it was placed in an ultra-low temperature freezer. The serum calcium was measured with o-cresolphthalein complexone using a colorimetric method (oCPC, Clinimate CA, Sekisui Medical, Tokyo, Japan). In women, the normal range for serum calcium is 8.7–10.3 mg/dL. Electrochemiluminescence immunoassay was used to measure intact parathyroid hormone (iPTH) (ECLIA, Elecsys PTH, Roche, Indianapolis, IN, USA). For women, the normal range for iPTH is 15–65 pg/mL. The serum 25(OH)D levels were evaluated by an automated direct competitive chemiluminescent immunoassay (CLIA) kit that is capable of detecting both 25-hydroxyvitamin D2 and 25-hydroxyvitamin D3 (ADVIA Centaur Vitamin D Total, Siemens Healthcare, Munich, Germany). This assay was standardized to LC-MS/MS [26]. In this study, vitamin D deficiency was defined as a serum 25(OH)D level < 20 ng/mL based on the criteria of the Institute of Medicine (IOM) and the Endocrine Society of the USA [27,28]. An automated CLIA kit, specifically the Access Ostase Bone Specific Alkaline Phosphatase kit from Beckman in the USA, was used to measure the bone alkaline phosphatase (BAP) levels. The normal range for the BAP levels in postmenopausal women is ≤22.0 µg/L. A urine sample was obtained for evaluation of N-telopeptide type I collagen (NTX), and 10 mL of it was poured into a conical tube. The tube was placed in a refrigerator (4 °C). The sample was analyzed for NTX using a CLIA method with the VITROS Immunodiagnostic Products NTX Reagent Pack (Ortho-Clinical Diagnostics, Raritan, NJ, USA). The results are reported as nM BCE/mM Cr, representing the nanomoles of bone collagen equivalents per liter (nM BCE/L) divided by the millimoles of creatinine per liter (mM Cr/L). In postmenopausal women, the normal range for NTX is 26–124 nM BCE/mM Cr.

To measure areal BMD (g/cm^2^), a dual-energy X-ray absorptiometry instrument (ARIA BHR-1-76, GE Healthcare, Chicago, IL, USA) was utilized to examine the posterior-anterior lumbar spine (vertebrae L1-L4) and femoral hip (femoral neck and total hip).

### 2.5. Statistical Analysis

The participants’ general characteristics and lifestyle, sun exposure time, biochemistry, BMD, and nutrient intake were expressed as frequencies (%) or means ± standard errors (S.E). We tested for differences in variable results in summertime and wintertime. For normality of data, the Kolmogorov–Smirnov test was used to test the differences in the paired values of each variable. The paired *t*-test and Wilcoxon signed-rank test were conducted for regularly normalized variables and nonparametric variables, respectively. Pearson’s correlation between BMD and BMI and biochemical indices was used for verification. As the serum 25(OH)D levels did not meet the normality test, the serum 25(OH)D levels by vitamin D supplement intake were tested using the Mann–Whitney U test. The levels of serum 25(OH)D required to reach plateau iPTH levels were determined using the exponential decay model as follows [29,30]:

$$\mathrm{iPTH}\;=a+b\times \exp\left(c\times 25\left(\mathrm{OH}\right)D\right)$$

To investigate the associations between the annual average serum 25(OH)D levels and BMD, the participants were categorized into either a group below the criteria or a group above the criteria of the IOM vitamin D deficiency cut-off (20 ng/mL) [27] and the serum 25(OH)D level to achieve the plateau PTH concentration obtained in this study (18.9 ng/mL). Thereafter, linear regression analysis was performed with YSM, BMI, vitamin D intake/1000 kcal, calcium intake/1000 kcal, and BAP as covariates. To examine the lumbar spine (L-spine) BMD by the levels of calcium and vitamin D intake, an analysis of covariance (ANCOVA) was performed. For the purposes of this examination, the participants were categorized into four groups, on the basis of high and low levels of vitamin D and calcium, according to the median vitamin D and calcium intake per 1000 kcal. The median vitamin D and calcium intakes per 1000 kcal during the summertime were 5.21 µg and 342.5 mg, respectively. The median vitamin D and calcium intakes per 1000 kcal during the wintertime were 5.53 µg and 353.2 mg, respectively. The median annual averages of the vitamin D and calcium intakes per 1000 kcal were 7.11 µg and 343.0 mg, respectively. The four groups included the low vitamin D-low calcium group (LDLC), low vitamin D-high calcium group (LDHC), high vitamin D-low calcium group (HDLC), and high vitamin D-high calcium group (HDHC). The covariates were the YSM, BMI, and NTX.

To analyze the variables affecting the serum 25(OH)D levels, the stepwise method in the multivariate linear regression analysis was performed. Age, drinking status, sun exposure time, number of ways to avoid sun exposure, IPAQ, BMI, and vitamin D intake/1000 kcal were input variables. The *p*-values > 0.10 were used to exclude variables. To evaluate the vitamin D level required to maintain the serum 25(OH)D levels at 18.9 ng/mL and 20 ng/mL, receiver operating characteristic (ROC) analysis was performed. The ROC curve graph shows cut-off concentrations of serum 25(OH)D calculated using Youden’s index formula, *p*-values, and the area under the ROC curve (AUC).

For statistical analysis, SPSS 28.0 software (IBM Corp., Armonk, NY, USA) was used. MedCalc^®^ Statistical Software (MedCalc Software Ltd., Ostend, Belgium; Version 20.216) was used for comparison of areas under the ROC curves. Exponential decay was presented with GraphPad Prism 9 (GraphPad Software, Inc., San Diego, CA, USA; Version 9.5.1). Statistical significance was defined using a *p*-value < 0.05, and all tests were conducted with a two-sided approach.

## 3. Results

### 3.1. General Characteristics and Lifestyle of the Participants

Table 1 shows the baseline general characteristics of the 96 participants who participated in all study procedures for a year. The participants’ average age was 57.08 years, and that of the YSM was 7.27 years. Most participants (60.4%) indicated that they used three or more ways to avoid sun exposure. The proportion of participants who did not drink was 64.6%. Most participants (76.0%) indicated that they had moderate-intensity physical activity.

There were 50 (52.1%) and 53 (55.2%) participants who took vitamin D supplements during summertime and wintertime, respectively. There were 25 (26.0%) and 34 (35.4%) participants who took calcium supplements during summertime and wintertime, respectively. Sun exposure time from 10 a.m. to 3 p.m. was 35.95 ± 24.63 min and 38.23 ± 28.46 min during summertime and wintertime, respectively. There was no significant difference observed between the seasons.

### 3.2. Body Mass Index, Biochemical Indices, and Bone Mineral Density by Season

**Figure 1:**
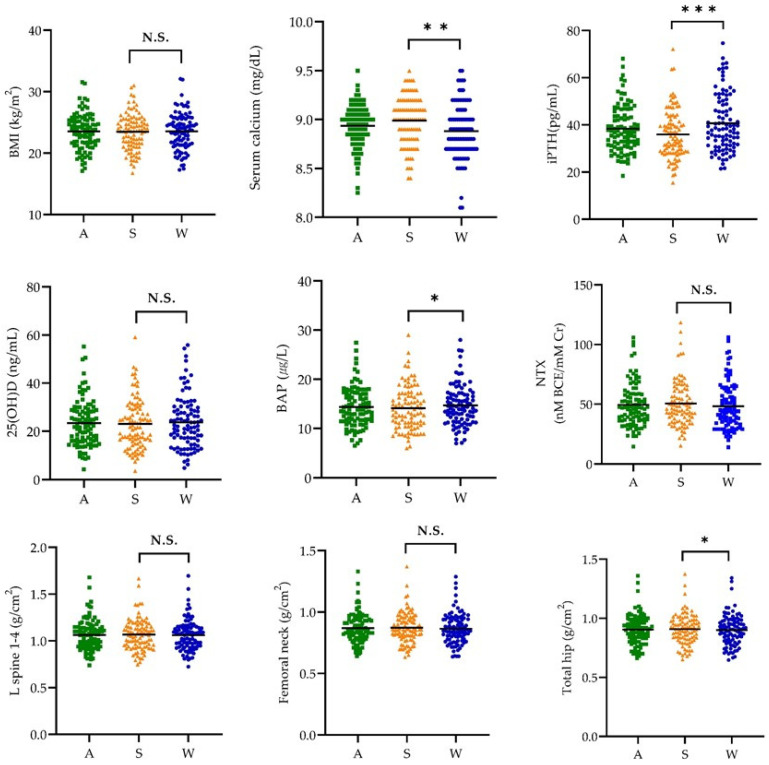
Body mass index, biochemical indices, and bone mineral density by season (*n* = 96). Solid line: mean value; A, annual average; S, summertime; W, wintertime; BMI, body mass index; iPTH, intact parathyroid hormone; BAP, bone alkaline phosphatase; NTX, type Ι collagen cross-linked N-telopeptide; BMD, bone mineral density. The *p*-values for the serum calcium and serum 25(OH)D levels were derived from the Wilcoxon signed-rank test. The *p*-values for BMI, iPTH, BAP, NTX, and BMD were derived from the paired *t*-test. The *p*-values for the serum calcium and serum 25(OH)D levels were derived from the Wilcoxon signed-rank test. *** *p* < 0.001, ** *p* < 0.01, * *p* < 0.05. N.S.: not significant.

Figure 1 presents the scatter plot of the BMI, biochemical indices, and BMD by season.

The mean BMI values were not significantly different between summertime and wintertime. Among the biochemical indices, the serum calcium levels were significantly higher in the summertime than in the wintertime (8.99 ± 0.24 vs. 8.88 ± 0.28 mg/dL, *p* < 0.01). In contrast, the wintertime values of iPTH (40.72 ± 12.02 vs. 35.97 ± 10.36 pg/mL, *p* < 0.001) and BAP (14.66 ± 4.11 vs. 14.12 ± 4.39 µg/L, *p* < 0.05) were significantly higher than those in the summertime. No significant differences in the mean serum 25(OH)D (23.12 ± 1.05 vs. 23.80 ± 1.11 ng/mL) and NTX (50.56 ± 1.93 vs. 48.26 ± 2.01 nM BCE/mM Cr) levels were observed between the two seasons. The mean BMD of the total hip was significantly higher in the summertime (0.91 ± 0.01 vs. 0.90 ± 0.01 g/cm^2^, *p* < 0.05), whereas the L-spine 1–4 (1.07 ± 0.02 vs. 1.06 ± 0.02 g/cm^2^) and femoral neck (0.87 ± 0.01 vs. 0.86 ± 0.01 g/cm^2^) showed no significant differences.

### 3.3. Correlation Coefficient of Body Mass Index, Biochemical Indices, and Bone Mineral Density

Table 2 shows Pearson’s correlation coefficient of BMD of the L-spine 1–4, femoral neck, and total hip; BMI; and biochemical indices in the summertime, the wintertime, and the annual average. There was a significant positive correlation between the annual average BMI and BMD in all skeletal sites (L-spine *p* < 0.05, femoral neck *p* < 0.01, total hip *p* < 0.001). Additionally, the NTX and BMD levels showed a significant negative correlation only in the L-spine. In the summertime, no biochemical indices showed a significant correlation with the BMD. However, the BMI showed a positive correlation with the BMD at all skeletal sites (L-spine, femoral neck *p* < 0.05, total hip *p* < 0.001). In the wintertime, the BMDs of the L-spine 1–4 and femoral neck showed a significant negative correlation with the NTX and BMD levels, respectively. The BMD of the total hip showed a significant negative correlation with the NTX (*p* < 0.05) and BAP (*p* < 0.001) levels. Additionally, in the wintertime, the BMI was positively correlated with the BMDs of all the skeletal sites (L-spine, *p* < 0.05; femoral neck, *p* < 0.01; total hip, *p* < 0.001).

### 3.4. Nutrient Intake

Table 3 shows the results of the mean daily intake of some nutrients. The mean energy intakes were 1623.38 ± 36.54 kcal/day and 1736.27 ± 49.10 kcal/day in the summertime and wintertime, respectively, showing that energy intake was significantly higher in the wintertime. No significant differences in carbohydrate, fat, protein, calcium, and vitamin D intakes by 1000 kcal energy were observed between the two seasons. Although the total calcium intake per 1000 kcal was higher in the wintertime than in the summertime, it was not statistically significant. In total, 25 and 34 participants took calcium supplements in the summertime and wintertime, respectively. Calcium intake, including supplements, was 529.61 ± 22.43 mg/1000 kcal/day in the summertime and 549.89 ± 28.00 mg/1000 kcal/day in the wintertime. This was significantly higher than the calcium intake (summertime, 316.56 ± 9.99 mg/1000 kcal/day; wintertime, 314.67 ± 9.74 mg/1000 kcal/day) of individuals who did not take calcium supplements (summertime *n* = 71; wintertime *n* = 62) (*p* < 0.001). Fifty and 53 participants took vitamin D supplements in the summertime and wintertime, respectively. Vitamin D intake, including supplements, was 16.52 ± 2.13 µg/1000 kcal/day and 18.79 ± 2.23 µg/1000 kcal/day in the summertime and wintertime, respectively. However, the vitamin D intake of individuals (summertime *n* = 46; wintertime *n* = 43) who did not take vitamin D supplements was 3.06 ± 0.24 µg/1000 kcal/day in the summertime and 2.53 ± 0.25 µg/1000 kcal/day in the wintertime, which was significantly lower than that of individuals taking supplements (*p* < 0.001).

### 3.5. Serum 25(OH)D Level According to Vitamin D Supplementation

**Figure 2:**
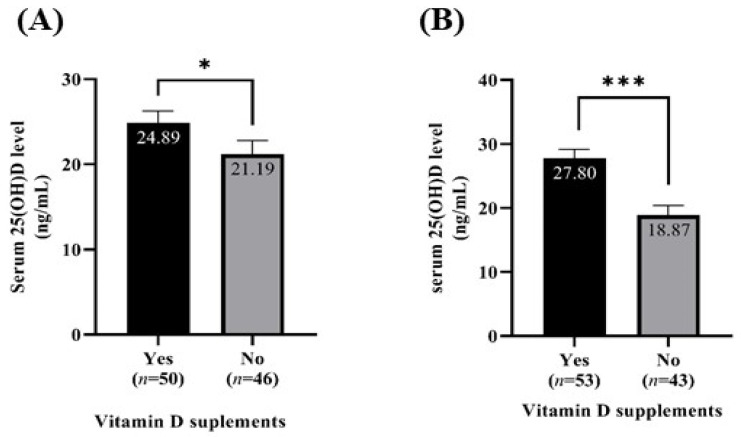
The serum 25(OH)D level according to vitamin D supplementation. (**A**) Summertime; (**B**) Wintertime. Mean ± S.E.; The mean value of vitamin D intake through vitamin D supplements: summertime, 22.61 ± 3.09 µg/day; wintertime, 24.84 ± 3.53 µg/day. The *p*-values were derived from the Mann–Whitney U test.; * *p* < 0.05, *** *p* < 0.001.

Based on whether they took vitamin D supplements or not, the participants were categorized into two groups to compare their serum 25(OH)D levels (Figure 2). For participants who took vitamin D supplements, the mean daily vitamin D intake through supplements was 22.61 and 24.84 µg/day in the summertime and wintertime, respectively. The serum 25(OH)D levels in the summertime (24.89 ± 1.35 vs. 21.19 ± 1.61 ng/mL, *p* < 0.05) and wintertime (27.80 ± 1.35 vs. 18.87 ± 1.54 ng/mL, *p* < 0.001) were significantly higher in those who took supplements.

### 3.6. Exponential Decay for Optimal Serum 25(OH)D to Suppress iPTH Elevation

**Figure 3:**
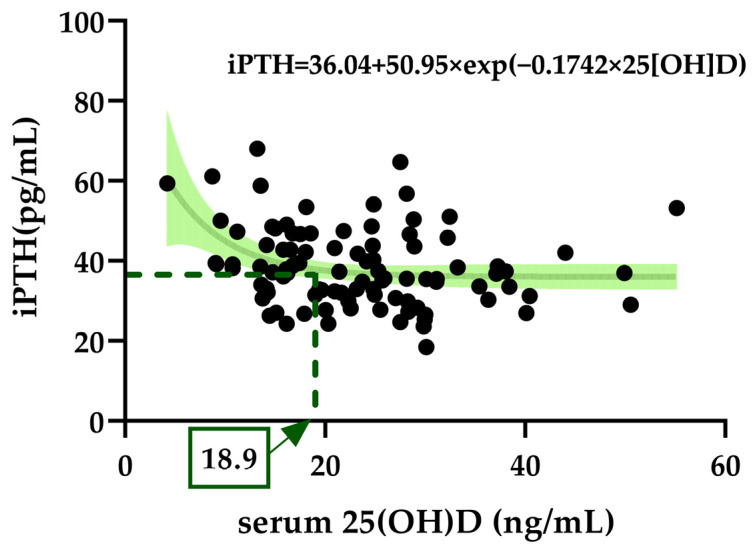
Exponential decay for optimal serum 25(OH)D to suppress iPTH elevation. The 25(OH)D level at which iPTH was elevated was an annual average of ≤18.9 ng/mL.

An exponential decay function was used to calculate the relationship between changes in the iPTH and serum 25(OH)D levels in the annual average, revealing that the minimum serum 25(OH)D concentration required to reach a plateau in iPTH concentration was 18.9 ng/mL (Figure 3). The exponential decay curve fit the following equation:

$$\mathrm{iPTH}=36.04+50.95\times \exp\left(-0.1742\times 25\left(\mathrm{OH}\right)D\right)$$

### 3.7. Relationship between Bone Mineral Density and Serum 25(OH)D Levels

Table 4 presents the categorization of participants’ department into two groups, based on whether their annual average serum 25(OH)D levels were below or above two cut-off points: the reference value for vitamin D deficiency (20 ng/mL), recommended by the IOM and Endocrine Society of the USA [27,28], and the optimal serum 25(OH)D level (18.9 ng/mL) identified in this study to suppress iPTH elevation. The correlation between the BMD of skeletal sites and the serum 25(OH)D levels were analyzed for each group. In both cases, groups with serum 25(OH)D levels lower than the cut-off values showed a positive correlation between the femoral neck and total hip BMDs and the serum 25(OH)D levels.

### 3.8. L-Spine Bone Mineral Density According to Calcium and Vitamin D Intake

**Figure 4:**
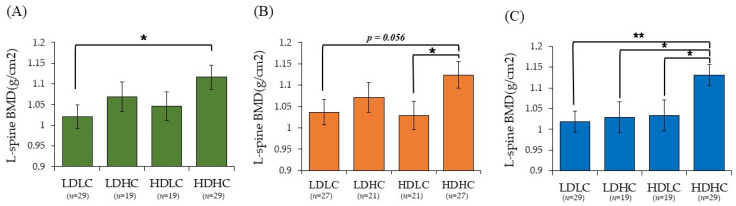
L-spine bone mineral density according to calcium and vitamin D intake. (**A**) Annual average; (**B**) Summertime; (**C**) Wintertime. Intake of vitamin D and calcium per 1000 kcal in the annual average, summertime, and wintertime was divided by the median value (Annual: vitamin D/1000 kcal: median, 7.11 µg; calcium/1000 kcal: median, 342.96 mg; Summertime: vitamin D/1000 kcal: median, 5.21 µg; calcium/1000 kcal: median, 342.48 mg; Wintertime: vitamin D/1000 kcal: median, 5.53 µg; calcium/1000 kcal: median, 353.22 mg). Covariates: YSM, BMI, NTX. The *p*-values were derived from ANCOVA. * *p* < 0.05, ** *p* < 0.01.

According to the results shown in Table 4, the BMD of the L-spine was not significantly correlated with the serum 25(OH)D levels. In accordance with a previous study, the L-spine is known to be sensitive to environmental change and has a rapid bone turnover rate [31]. Therefore, we analyzed the BMD of the L-spine using calcium and vitamin D intake (Figure 4). The BMD of the L-spine in the four groups was compared. The annual average BMD of the L-spine was significantly higher in the HDHC group than that in the LDLC group (Figure 4A, *p* < 0.05).

In the summertime, the BMD of the L-spine was significantly higher in the HDHC group than that in the HDLC group (Figure 4B, *p* < 0.05). In the wintertime, the BMD of the L-spine was significantly higher in the HDHC group than that in all other groups (Figure 4C, LDHC and HDLC groups: *p* < 0.05; LDLC group: *p* < 0.01).

### 3.9. Multivariate Linear Regression Analyzing the Factors Influencing the Serum 25(OH)D Level by Season

The analysis was conducted using multiple linear regression for variables affecting the annual average serum 25(OH)D levels, and vitamin D intake/1000 kcal, BMI, and sun exposure time were significant influencing factors (Table 5). For every 1 µg/1000 kcal increase in vitamin D intake, there was a significant rise in the serum 25(OH)D levels by 0.370 ng/mL (*p* < 0.001). The serum 25(OH)D levels decreased by 0.928 ng/mL (*p* < 0.01) with increasing BMI, while, on the other hand, the serum 25(OH)D levels increased by 0.105 ng/mL (*p* < 0.05) with each additional minute of sun exposure. Factors affecting the serum 25(OH)D levels were vitamin D intake, sun exposure time, and BMI in the summertime and vitamin D intake and BMI in the wintertime. When the intake of vitamin D raised by 1 µg/1000 kcal, there was an increase of 0.226 ng/mL and 0.314 ng/mL in the serum 25(OH)D levels during summertime and wintertime, respectively (*p* < 0.01, *p* < 0.001, respectively). In the summertime, an increase of 1 min in sun exposure time led to a rise of 0.100 ng/mL in the serum 25(OH)D levels (*p* < 0.05). As the BMI increased, the serum 25(OH)D levels decreased by 0.674 and 0.985 ng/mL in the summertime and wintertime, respectively (*p* < 0.05, *p* < 0.01, respectively).

### 3.10. ROC Analysis for Optimal Vitamin D Intake at Serum 25(OH)D ≥20 and ≥18.9 ng/mL

**Figure 5:**
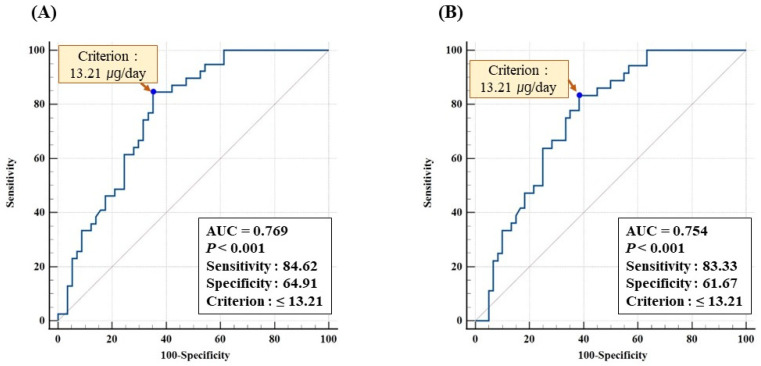
Receiver operating characteristic (ROC) analysis for optimal vitamin D intake at serum 25(OH)D ≥20 ng/mL (**A**) and ≥18.9 ng/mL (**B**). AUC, area under the ROC curve.

To maintain serum 25(OH)D levels of at least 20 ng/mL, the required daily vitamin D intake was identified through ROC analysis, with an adequate intake criterion of 13.21 µg/day (AUC = 0.769, *p* < 0.001, Figure 5). Furthermore, the vitamin D intake criterion analyzed using ROC, based on the optimal serum 25(OH)D derived in this study (18.9 ng/mL), was also 13.21 µg/day (AUC = 0.754, *p* < 0.001).

## 4. Discussion

Postmenopausal women are at increased risk for osteopenia, osteoporosis, and bone fractures, which reduce the quality of life of older adults [9]. Since 2008, the Korean government has conducted the Korea National Health and Nutrition Examination Survey (KNHANES) to measure serum vitamin D concentrations, which provides a foundation for research on the status of vitamin D levels in Korea. Additionally, a food composition database for vitamin D among the foods consumed by Koreans was officially published in 2016 via the 9th Korea National Standard Food Composition Table. Research on vitamin D in Republic of Korea was launched later than it was in the USA [32] and European countries [33]. As we were concerned regarding the vitamin D status in Koreans, we began constructing a database for vitamin D internally in 2009. The data were used to reanalyze KNHANES, and it showed that compared to women aged 30–49 years, vitamin D intake in Korean women aged 50–64 years was lower or similar, but the serum 25(OH)D levels were higher [13,14]. However, since the KNHANES has not conducted BMD examinations since 2011 and has not measured the serum 25(OH)D levels since 2014, vitamin D-related studies have to be conducted individually in Republic of Korea.

Accordingly, this study on the vitamin D status of Korean postmenopausal women was carried out subsequent to a study that identified the optimal vitamin D concentrations in young Korean women [34]. Furthermore, this study aims to compare the variables that affect vitamin D status, as collected from previous studies conducted on young Korean women, with the findings of the current study. In previous studies of women in their 20s who had not reached peak bone mass, it was difficult to identify correlations between BMD and vitamin D status. This study aimed to assess the correlations between BMD and vitamin D status in postmenopausal women with decreasing bone mass.

The mean serum 25(OH)D level of participants in this study was 23.46 ± 0.99 ng/mL, which was higher than the serum 25(OH)D levels in the summertime in young Korean women in their 20s (17.9 ± 0.61 ng/mL) [34]. This is the same result as that of previous studies, using data from KNHANES, in which serum vitamin D concentrations were higher in women aged ≥50 years than in those aged <50 years [13,14,15]. In older adults, the serum 25(OH)D levels are known to be reduced owing to skin aging [12]. Postmenopausal women have a disadvantaged vitamin D metabolism because their body fat is higher than that of young women. The serum 25(OH)D levels are negatively correlated with body fat mass as the majority of 25(OH)D is stored in adipose tissue, leading to decreased circulating serum 25(OH)D levels [35]. Especially, the serum 25(OH)D levels in older women are highly likely to be reduced. However, not only in Asian countries, such as Republic of Korea, Turkey [36], Taiwan [37], Thailand [16], and Japan [17], but also in Canada [38], older adults were reported to exhibit higher levels of serum 25(OH)D than young adults. Accordingly, it was necessary to investigate factors affecting the serum 25(OH)D levels for each group.

Factors affecting the serum 25(OH)D levels in this study and the previous study on young Korean women in their 20s were compared [34]. Compared to young Korean women, postmenopausal women used more means to avoid sun exposure, but this was not the variable affecting the serum 25(OH)D levels. However, postmenopausal women were exposed to UV radiation approximately 2.6 and 1.9 times longer than young Korean women in their 20s (14.5 min in the wintertime and 19.5 min in the summertime) [34]. Additionally, as sun exposure time increases by 1 min, the serum 25(OH)D levels also increase. Compared to those in women in their 20s (total participants [annual] 0.056 ng/mL; summertime 0.059 ng/mL) [34], the average levels of serum 25(OH)D in postmenopausal women were approximately 1.9-fold higher in the annual average data and 1.7-fold higher in the summertime data. This suggests that the increased total UV radiation exposure due to the increased time spent in outdoor activities is a major factor in increasing the serum 25(OH)D levels.

Moreover, vitamin D intake was found to be a variable affecting the serum 25(OH)D levels in both young women and postmenopausal women in Korea. The total intake of vitamin D, including vitamin D supplements, in young Korean women was 8.1 µg/day in the summertime and 7.4 µg/day in the wintertime [34]. That is just 49.1% and 39.3% of the total vitamin D intake in postmenopausal women in this research. The proportions of young Korean women taking vitamin D supplements were 30.8% in the summertime and 18.8% in the wintertime [34], which were lower than the proportions of postmenopausal women taking vitamin D supplements in this research (52.1% and 55.2%). The influence of vitamin D intake on the serum 25(OH)D levels in postmenopausal women were 2.2-fold, 1.5-fold, and 1.9-fold higher in the annual average, summertime, and wintertime, respectively, compared to that in young Korean women. Intake of vitamin D supplements can be considered one of the factors increasing the serum 25(OH)D levels in postmenopausal women.

Unlike the young Korean women in the previous study [34], BMI was one of the variables explaining the serum 25(OH)D levels in this study. The BMI of young Korean women ranged from ≥18 to <23 kg/m^2^. However, as body fat mass in postmenopausal women is mostly higher than that in young Korean women in their 20s, this study included women with BMIs ≥ 18.5 and <30 kg/m^2^. We believe that the correlation between the serum 25(OH)D levels and BMI in young Korean women was not observed because of the smaller range of variation in the BMI in young Korean women. Postmenopausal women are at increased risk for obesity and bone loss, which can contribute to a condition known as osteosarcopenic obesity, characterized by the co-occurrence of obesity, sarcopenia, and osteoporosis. In osteosarcopenic obesity, known as a novel pathological condition characterized by a possible bidirectional link between vitamin D and muscle, the skeletal and adipose tissues may lead to the problem of vitamin D deficiency [39]. However, further research is needed to better understand the causes and consequences of this condition.

In conclusion, the serum 25(OH)D levels were higher in Korean postmenopausal women than in young women because of increased intake of vitamin D through supplements and increased sun exposure time. This result supports the findings of studies conducted on Asians, which reported that the incidence of serum 25(OH)D deficiency is lower in older than in young adults because young adults are likely to work indoors, while older adults are likely to work outdoors [12,18]. Additionally, in a Canadian study, the serum 25(OH)D levels were found to be higher in non-white older women than in young women because more non-white older women took vitamin D supplements [38]. This indicates that taking vitamin D supplements helps maintain the levels of vitamin D in the serum.

No difference in the serum 25(OH)D levels between summertime and wintertime was observed, which may be attributed to the intake of supplements. Three more women took vitamin D supplements in the wintertime compared to the summertime. In individuals taking supplements, the intake of vitamin D increased from 22.61 ± 3.09 in the summertime to 24.84 ± 3.53 µg/day in the wintertime. Compared to individuals not taking supplements, the serum 25(OH)D levels in women taking vitamin D supplements were higher in both summertime and wintertime. Especially in the wintertime, there was a significant difference in the serum 25(OH)D levels with or without supplements. Therefore, it is crucial to take adequate supplements to preserve the serum 25(OH)D levels in the winter season, when there is a decline in UV radiation levels.

No association between the serum 25(OH)D levels and age was found in postmenopausal women aged 45–69 years in this study. Despite the expectation of an increase in the serum 25(OH)D levels with age due to higher levels in postmenopausal women than young women, age was not identified as a variable affecting the serum 25(OH)D levels. Similar findings were reported in previous studies conducted in Korean [40], Chinese [41], and Japanese [42] postmenopausal women, indicating no correlation between age and serum 25(OH)D levels. Adequate vitamin D intake and UV radiation were found to be able to sustain the serum 25(OH)D levels even in the presence of increased body fat and reduced vitamin D3 synthesis in the skin.

Elevated levels of bone turnover markers (BTMs) indicate an accelerated rate of bone turnover, which can lead to decreased bone mass and an increased risk of bone fracture [43]. Therefore, this study investigated iPTH, NTX, and BAP of BTMs. Compared to NTX (30.2 nM BCE/mM Cr in summertime and 41.0 nM BCE/mM Cr in wintertime) and BAP levels (10.68 µg/L in summertime and 10.52 µg/L in wintertime) from a previous study involving young Korean women [34], both NTX and BAP levels in postmenopausal women were higher. Additionally, iPTH and NTX levels in young Korean women in the previous study were significantly higher in the wintertime than in the summertime [34]. In this study, the levels of iPTH and BAP were significantly higher in the wintertime compared to those in the summertime. BTMs were known to be 20–30% higher in the wintertime than those in the summertime [44]. Even though there was no significant correlation between BMD and BTMs in young Korean women, this study showed that the BMDs of the L-spine, femoral neck, and total hip were negatively correlated with NTX, BAP, and both NTX and BAP in the wintertime. This suggests that BTM as an index predicts changes in BMD in postmenopausal women, unlike that in young women.

Additionally, as a rapid increase in iPTH causes secondary hyperparathyroidism, a plateau value that does not rapidly increase an iPTH level should be used when defining the optimal serum 25(OH)D levels [29,30]. In this investigation, the iPTH level did not rapidly increase when the serum 25(OH)D level was 18.9 ng/mL. This was similar to the serum 25(OH)D level of 18.44 ng/mL in young Korean women from the previous study [34]. A previous study by Choi et al. in Korean women aged ≥50 years demonstrated optimal serum 25(OH)D levels by using a plateau that does not rapidly increase the PTH level. This study reported that the optimal serum 25(OH)D level was 13.8 ng/mL [45]. Lee et al. reported an optimal serum 25(OH)D level of 20 ng/mL in Korean postmenopausal women diagnosed with osteoporosis or osteopenia [46]. The optimal 25(OH)D level in Chinese postmenopausal women aged ≥55 years was reported to be 16.78 ng/mL [47]. The optimal serum 25(OH)D levels reported from the previous investigations were similar to or slightly lower than the values obtained from the participants in this research.

Based on the optimal serum 25(OH)D level (18.9 ng/mL), which does not increase the iPTH level, obtained from this study, the participants with lower serum 25(OH)D concentrations had a positive correlation between the BMDs of the femoral neck and total hip and serum 25(OH)D levels. According to a study by Hwang et al., which analyzed data from KNHANES in 2009, the BMD of the total hip significantly increased up to serum 25(OH)D levels of 20.4 ng/mL in adults aged ≥49 years. Above this level, no significant changes were observed, similar to the results of this study [48,49,50]. However, a study in Southwestern Asians reported that there was no association between BMD and serum 25(OH)D. As the study results are different, more epidemiological studies are necessary [51,52].

Moreover, this study did not identify correlations between the serum 25(OH)D levels and L-spine BMD. As the L-spine is mainly composed of trabecular bone, it has high metabolic activity and is sensitive to environmental changes inside the body [31]. Factors affecting the BMD can vary depending on the bone composition and structure, and the density of the L-spine with dynamic metabolic activity may change depending on dietary factors. In an investigation of Korean postmenopausal women, a group with a diet rich in dairy products and fruit had the highest L-spine BMD [53]. In a double-blind, randomized, placebo-controlled study conducted in Denmark for 2 years among postmenopausal women, L-spine BMD increased by 1.6% in healthy postmenopausal women who took vitamin D and calcium supplements, but no differences in the distal forearm and femoral neck were observed [54]. This study investigated the correlations between nutrient intake and BMD and observed a significant finding in the L-spine which has high metabolic activity. The group with both vitamin D and calcium intake levels above the medians was the most beneficial for L-spine BMD. The recommended calcium nutrient intake (RNI) for women aged ≥50 years by KDRIs was 800 mg/day [23]. The participants’ calcium intake in this study was approximately 75.5–82.5% of the RNI by KDRIs. Among them, the total intake of calcium in individuals taking calcium supplements (25 in summertime and 34 in wintertime) was 107.4 and 119.3% in the summertime and wintertime, respectively, compared to calcium RNI by KDRIs (data not presented). Calcium is a very important nutrient for postmenopausal women with decreased BMD as it forms and maintains the skeleton. However, during the menopausal years, calcium absorption decreases, and calcium release from the bone becomes more active [55]. Intake of dietary calcium in the participants of this study of Korean women was 450–500 mg/day [56], which is lower than the RNI but is not easy to improve. Maintaining an adequate level of vitamin D in the body can enhance calcium absorption in the intestine and prevent bone loss by suppressing the rise of serum PTH, which can result in an elevation in BMD [2,10]. In this study, the LDHC group, with low vitamin D intake and high calcium intake, in the wintertime observed a significantly lower L-spine BMD compared to the HDHC group, with high calcium and vitamin D intakes. This suggests that L-spine BMD is more dependent on vitamin D intake than calcium intake during the period when solar radiation is low. A previous study also demonstrated that vitamin D intake affects the BMD in the wintertime when solar radiation is low in the northern hemisphere [57]. Therefore, it is necessary to take adequate amounts of calcium and vitamin D for healthy bones.

An intake of 13.21 µg/day of vitamin D was required to sustain optimal serum 25(OH)D levels >18.9 ng/mL and 20 ng/mL. KDRIs are updated every 5 years, and the vitamin D AI in individuals aged ≥50 years in 2010 was 10 µg/day, which was changed to 10 and 15 µg/day for those aged <65 and ≥65 years, respectively, in 2015 [23]. As the geographical location of the country, the lifestyle of the population, and vitamin D intake levels are different, each country and expert has different guidelines for vitamin D intake [58]. Nevertheless, as a limited amount of research has been conducted on optimal vitamin D intake in Koreans, the vitamin D AI was established using the results of studies conducted in other countries. Based on the results of KNHANES between 2009 and 2014 [13,14,15], the previous study in young Korean women in their 20s [34], and the results of this study, Korean postmenopausal women consistently exhibited a higher serum 25(OH)D level compared to that before menopause. Considering the physiological changes in postmenopausal women (bone resorption accompanied by a decline in estrogen levels, decreased vitamin D3 synthesis in the epidermis, and an inverse correlation between BMI and serum 25[OH]D) and the average menopausal age of Korean women (49.9 years) [59], it seems appropriate to reset the vitamin D AI to 15 μg/day for Korean women aged ≥50 years.

In this study, the participants had an annual average daily vitamin D intake of 17.65 ± 1.75 µg, which exceeded the vitamin D AI recommended by the KDRIs. High vitamin D intake in this study resulted from the intake of supplements. The vitamin D intake of the group not taking vitamin D supplements was only 4.96 µg/day in the summertime and 4.47 µg/day in the wintertime. Fish and shellfish were the highest vitamin D contributors at 57%, among the vitamin D source foods consumed by the participants (Figure S1).

As the foods that are sources of vitamin D consumed by Koreans are not diverse, it is challenging for them to achieve the vitamin D AI of KDRIs by obtaining vitamin D from the natural diet alone. Therefore, when considering serum 25(OH)D levels and bone health in postmenopausal women, taking vitamin D-fortified food or vitamin D supplements is necessary. For fortified foods, a vitamin D fortification of approximately 5 μg per serving of food is considered adequate, similar to the amount suggested in our previous study [34]. This amount of fortification is safe and would not exceed 100 µg/day, the UL of KDRI for postmenopausal women, even considering duplication of vitamin D intake from supplements and fortified food. A policy that fortifies foods that are frequently consumed by Koreans with vitamin D should be tested for its effects after implementation.

There are some limitations to this study. First, due to the COVID-19 pandemic, the participants’ lifestyle factors, including outdoor time, sun exposure time, and dietary intake, may have been affected, as this study was conducted during this period. Second, as this study only included a small sample of women residing in a metropolitan area of Republic of Korea, the findings cannot be generalized to all postmenopausal women in Korea. Therefore, future studies, including a more diverse population, are needed.

Notwithstanding these limitations, our study has several advantages. First, this is the first study to examine the vitamin D intake of Koreans and establish a basis for vitamin D AI levels for dietary reference intakes in Korea. Second, we have determined the optimal serum 25(OH)D concentrations and intakes for Korean postmenopausal women, who are most vulnerable to bone disease. Finally, our study compared the results with those of a previous investigation performed on young Korean women, and the findings confirmed that higher serum 25(OH)D levels in older Southeast Asian and Korean women were attributed to the increased intake of vitamin D supplements and outdoor time.

## 5. Conclusions

In postmenopausal women, several factors have been identified as influencing serum 25(OH)D levels, in particular vitamin D intake, sun exposure time, and BMI in the annual average and summertime, and vitamin D intake and BMI in the wintertime. In comparison to young Korean women in their 20s, postmenopausal women exhibit higher vitamin D intake and are engaged in more outdoor activities. These factors are considered to be two crucial determinants of serum 25(OH)D levels in postmenopausal women. The serum 25(OH)D level that does not lead to a rapid increase in the iPTH level in postmenopausal women is 18.9 ng/mL. In the group with serum 25(OH)D level <18.9 ng/mL, the BMDs of the femoral neck and total hip were positively correlated with the serum 25(OH)D level. Therefore, maintaining the serum 25(OH)D levels at ≥18.9 ng/mL is considered beneficial for bone health. Additionally, in the group with calcium and vitamin D intake higher than the corresponding medians, L-spine BMD was significantly higher. The results were obvious in the wintertime when UV radiation was low. In this study, the vitamin D intake that maintained serum 25(OH)D levels at ≥18.9 ng/mL was 13.21 µg/day. It will be desirable to change the current vitamin D AI of KDRIs to 15 µg/day for Korean women aged ≥50 years. For the bone health of postmenopausal women, selecting foods and supplements rich in calcium and vitamin D will be helpful. However, it is difficult to reach the vitamin D intake of 13.21 µg/day, as recommended by this study, by only consuming natural foods. Therefore, we would like to suggest a policy to fortify foods that are often consumed by Koreans with approximately 5 µg of vitamin D per serving.

## Figures and Tables

**Table 1:** General characteristics and lifestyle of the participants.

Variables		Mean ± S.E or N (%)
N		96 (100.0)
Age (years)		57.08 ± 0.55
YSM (years)		7.27 ± 0.57
Height (cm)		158.2 ± 0.51
Weight (kg)		58.42 ± 0.85
Number of ways to avoid sun exposure	0	9 (9.4)
	1	9 (9.4)
	2	20 (20.8)
	≥3	58 (60.4)
	Subtotal	96 (100.0)
Alcohol consumption	Yes	34 (35.4)
	No	62 (64.6)
	Subtotal	96 (100.0)
IPAQ	High	6 (6.3)
	Moderate	73 (76.0)
	Low	17 (17.7)
	Subtotal	96 (100.0)
Taking vitamin D supplements	Summertime	50 (52.1)
	Wintertime	53 (55.2)
Taking calcium supplements	Summertime	25 (26.0)
	Wintertime	34 (35.4)
sun exposure time (min)	Summertime	35.95 ± 2.59
	Wintertime	38.23 ± 2.91 ^N.S.^

^N.S.^, Not significant compared to summertime using the paired *t*-test; YSM, Years since menopause. For ways to avoid sun exposure, participants were asked to select multiple responses from the following choices: use of sunscreen products, such as sunscreen lotion and cosmetics; hat, mask, or parasol with UV blocking effect; or other methods. Alcohol consumption: no, at least 6 months of abstinence from alcohol. IPAQ: “High” included individuals (1) who had high activity for 3 or more days, achieving a minimum of 1500 metabolic equivalents of task (MET)-min/week or (2) who had any combination of walking, moderate-intensity, or high-intensity activities for 7 or more days, achieving a minimum of 3000 MET-min/week. “Moderate” included individuals (1) who had high activity for at least 20 min a day for 3 or more days, (2) who had moderate-intensity activities or walking for at least 30 min a day for 5 or more days, or (3) who had any combination of walking, moderate-intensity or high-intensity activities for 5 or more days, achieving a minimum of 600 MET-min/week. “Low” (with the lowest level of physical activity) included individuals who did not respond to this item or who did not meet the criteria for the other two groups. Sun exposure time: 10 a.m.–3 p.m.

**Table 2:** Correlation coefficients of body mass index, biochemical indices, and bone mineral density.

	Annual Average			Summertime			Wintertime		
	L-Spine 1–4	Femoral Neck	Total Hip	L-Spine 1–4	Femoral Neck	Total Hip	L-Spine 1–4	Femoral Neck	Total Hip
BMI	0.245 *	0.306 **	0.398 ***	0.213 *	0.262 *	0.379 ***	0.255 *	0.323 **	0.397 ***
iPTH	−0.088	−0.161	−0.144	−0.120	−0.147	−0.176	−0.041	−0.138	−0.100
BAP	−0.174	−0.180	−0.196	−0.139	−0.143	−0.155	−0.198	−0.214 *	−0.233 **
NTX	−0.205 *	−0.150	−0.197	−0.153	−0.114	−0.123	−0.236 *	−0.152	−0.230 *
25(OH)D	−0.090	−0.131	−0.154	−0.112	−0.124	−0.150	−0.057	−0.091	−0.129
serumcalcium	0.139	−0.011	0.001	0.123	−0.035	−0.007	0.337	0.879	0.882

BMI, body mass index; iPTH, intact parathyroid hormone; BAP, bone alkaline phosphatase; NTX, type Ι collagen cross-linked N-telopeptide. The *p*-values were derived from Pearson’s correlation.; * *p* < 0.05, ** *p* < 0.01, *** *p* < 0.001.

**Table 3:** Nutrient intake.

		Nutrients per 1000 kcal (*n* = 96)			
Variables		Summertime	Wintertime	Annual Average	*p*-Value
Energy (kcal/day)		1623.38 ± 36.54	1736.27 ± 49.10	1679.83 ± 35.64	0.024
Carbohydrate (g/1000 kcal/day)		135.40 ± 1.74	136.11 ± 1.93	135.75 ± 1.48	0.872
Protein (g/1000 kcal/day)		44.36 ± 0.68	43.11 ± 0.55	43.73 ± 0.45	0.135
Fat (g/1000 kcal/day)		31.41 ± 0.54	31.27 ± 0.66	31.34 ± 0.48	0.530
Calcium total (mg/1000 kcal/day) ^†^		372.04 ± 13.40	397.98 ± 16.39	385.01 ± 12.50	0.136
	With supplements	529.61 ± 22.43	549.89 ± 28.00		
	Without supplements	316.56 ± 9.99	314.67 ± 9.74		
	*p*-value	<0.001	<0.001		
Vitamin D total (µg/1000 kcal/day) ^‡^		10.07 ± 1.31	11.51 ± 1.48	10.79 ± 1.07	0.371
	With supplements	16.52 ± 2.13	18.79 ± 2.23		
	Without supplements	3.06 ± 0.24	2.53 ± 0.25		
	*p*-value	<0.001	<0.001		

^†^ Calcium total: participants taking calcium supplements (summertime, *n* = 25, wintertime, *n* = 34); participants not taking calcium supplements (summertime, *n* = 71, wintertime, *n* = 62). ^‡^ Vitamin D total: participants taking vitamin D supplements (summertime, *n* = 50, wintertime, *n* = 53); participants not taking vitamin D supplements (summertime, *n* = 46, wintertime, *n* = 43). ^†^, ^‡^ Since supplement users were not paired, the Mann–Whitney U test was used to compare nutrient intake according to supplement use in each season. The *p*-values for energy (kcal/day) and protein (g/1000 kcal/day) were derived from the paired *t*-test. The *p*-values for carbohydrate (g/1000 kcal/day), fat (g/1000 kcal/day), total calcium (mg/1000 kcal/day), and total vitamin D (µg/1000 kcal/day) were derived from the Wilcoxon signed-rank test.

**Table 4:** Relationship between bone mineral density and the serum 25(OH)D levels.

Skeletal Site	Serum 25(OH)D Level	β	95% CI	*p*-Value
L-spine 1–4	<20 ng/mL ^†^ (*n* = 39)	0.004	−0.013, 0.021	0.639
	≥20 ng/mL (*n* = 57)	−0.002	−0.007, 0.004	0.595
Femoral neck	<20 ng/mL (*n* = 39)	0.012	0.001, 0.022	0.038
	≥20 ng/mL (*n* = 57)	−0.001	−0.005, 0.002	0.449
Total hip	<20 ng/mL (*n* = 39)	0.013	0.000, 0.026	0.050
	≥20 ng/mL (*n* = 57)	−0.001	−0.005, 0.002	0.378
L-spine 1–4	<18.9 ng/mL ^‡^ (*n* = 36)	0.006	−0.013, 0.024	0.532
	≥18.9 ng/mL (*n* = 60)	−0.001	−0.006, 0.004	0.673
Femoral neck	<18.9 ng/mL *(n* = 36)	0.015	0.004, 0.027	0.010
	≥18.9 ng/mL (*n* = 60)	0.000	−0.004, 0.003	0.784
Total hip	<18.9 ng/mL (*n* = 36)	0.018	0.005, 0.031	0.007
	≥18.9 ng/mL (*n* = 60)	−0.001	−0.004, 0.002	0.647

Covariates: YSM, BMI, vitamin D intake/1000 kcal, vitamin D intake/1000 kcal, BAP. The β coefficient is unstandardized in multivariate linear regression analysis. ^†^ 20 ng/mL is the deficiency concentration of serum 25(OH)D set by the Institute of Medicine (IOM). ^‡^ 18.9 ng/mL is the optimal concentration of serum 25(OH)D derived in this research.

**Table 5:** Multivariate linear regression analyzing the factors influencing the serum 25(OH)D level by season.

Seasons	Variables	β	95% CI	*p*-Value
Annual average	Vitamin D intake (µg/1000 kcal)	0.370	0.204, 0.536	<0.001
	BMI (kg/m^2^)	−0.928	−1.522, −0.334	0.003
	sun exposure time (min)	0.105	0.014, 0.195	0.024
Summertime	Vitamin D intake (µg/1000 kcal)	0.226	0.071, 0.381	0.005
	sun exposure time (min)	0.100	0.022, 0.178	0.013
	BMI (kg/m^2^)	−0.674	−1.318, −0.030	0.040
Wintertime	Vitamin D intake (µg/1000 kcal)	0.314	0.182, 0.446	<0.001
	BMI (kg/m^2^)	−0.985	−1.637, −0.334	0.003

The β coefficient is unstandardized in multiple linear regression analysis.; Vitamin D intake includes supplements.

## Data Availability

The data presented in this study are available on request from the corresponding author.

## Supplementary Materials

The following supporting information can be downloaded at: https://www.mdpi.com/article/10.3390/nu15081856/s1, Figure S1. Contribution of calcium (A) and vitamin D (B) intake by food source.
