# Supplementary material for: Optimal Serum 25(OH)D Levels and Vitamin D Intake in Korean Postmenopausal Women
Source: Nutrients. 2023 Apr 12;15(8):1856. doi: 10.3390/nu15081856 (PMC10144100; doi:10.3390/nu15081856)
Supplement: Supplementary file 1 [file nutrients-15-01856-s001.zip › nutrients-2332744-Supplementary material.pdf]

(A)

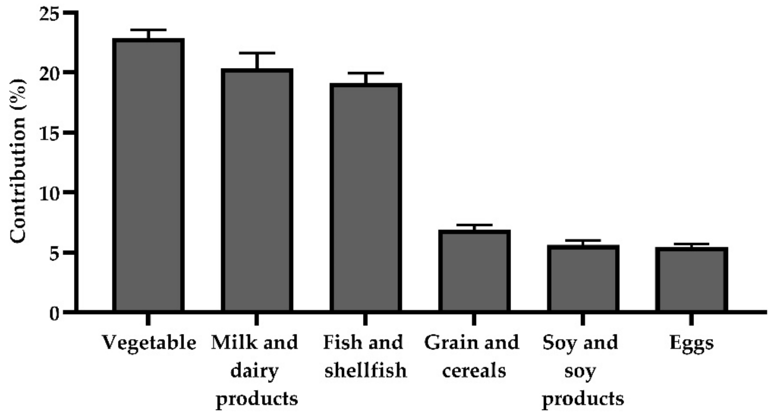

(B)

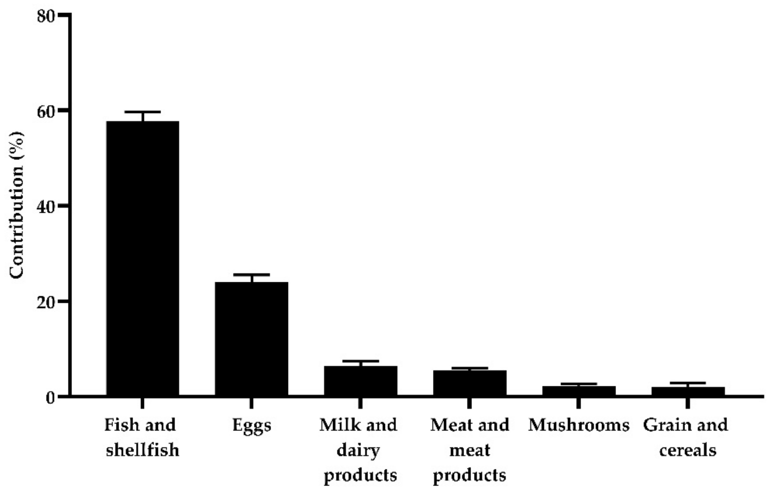

Figure S1. Contribution of calcium (A) and vitamin D (B) intake by food source. Mean ± S.E. Only the top 6 food groups for each nutrient are presented.
